# Genome Sequences of 11 Human Vaginal *Actinobacteria* Strains

**DOI:** 10.1128/genomeA.00887-16

**Published:** 2016-09-29

**Authors:** Amanda L. Lewis, Grace E. Deitzler, Maria J. Ruiz, Cory Weimer, SoEun Park, Lloyd S. Robinson, Kymberlie Hallsworth-Pepin, Aye Wollam, Makedonka Mitreva, Warren G. Lewis

**Affiliations:** a Department of Molecular Microbiology, Washington University School of Medicine, St. Louis, Missouri, USA; b Department of Obstetrics and Gynecology, Washington University School of Medicine, St. Louis, Missouri, USA; c Department of Medicine, Washington University School of Medicine, St. Louis, Missouri, USA; d Center for Women’s Infectious Disease Research, Washington University School of Medicine, St. Louis, Missouri, USA; e McDonnell Genome Institute, Washington University School of Medicine, St. Louis, Missouri, USA

## Abstract

The composition of the vaginal microbiota is an important health determinant. Several members of the phylum *Actinobacteria* have been implicated in bacterial vaginosis, a condition associated with many negative health outcomes. Here, we present 11 strains of vaginal *Actinobacteria* (now available through BEI Resources) along with draft genome sequences.

## GENOME ANNOUNCEMENT

Bacterial vaginosis (BV) is a vaginal dysbiosis associated with serious health complications (1–6). It is characterized by the absence of *Lactobacillus* species in the vagina and overgrowth of a polymicrobial community often containing members of the phylum *Actinobacteria*, including *Gardnerella vaginalis, Atopobium* spp., and others. In fact, *G. vaginalis* was recently shown to elicit several features of BV in a mouse vaginal infection model (7, 8). Bifidobacteria are also commonly isolated from the vagina, although members of this genus are rarely found in pathological contexts. Here, we isolated 11 vaginal bacteria from the phylum *Actinobacteria*. Vaginal swabs were collected from nonpregnant and pregnant women according to Washington University institutional review board (IRB)-approved protocols (201108155 and 20110382). Organisms isolated from vaginal swabs were cultured anaerobically, and identification was performed by 16S rRNA gene sequencing. Genomic DNA was obtained using the Wizard genomic DNA purification kit (Promega). Methodological details on isolation and clinical information will be described elsewhere.

Genomes were assembled *de novo* using the One Button Velvet assembly pipeline (version 1.1.06) (9) with hash sizes of 31, 33, and 35 after downsizing the sample input data to 100× coverage. An internal core gene screen on the assembly tested for completeness of the genome. After assembly, the minimum length for contigs was set to 200 bp, and an internal core gene screen was performed as defined by the Human Microbiome Project (HMP) (10). Then, adapters were removed, and low-quality regions were trimmed. Finally, a screen for contamination was performed. The process of gene annotation included generating both *ab initio* and evidence-based (BLAST) predictions. Functional predictions of coding sequences were made using GeneMark and Glimmer3 (11, 12). Loci were then defined by clustering predictions with the same reading frame. We evaluated predictions using the nonredundant (NR) and Pfam databases and resolved overlaps between adjacent coding genes. Intergenic regions not spanned by GeneMark and Glimmer3 were subject to a BLAST search against NCBI’s NR database and predictions generated based on protein alignments. tRNA genes were determined using tRNAscan-SE (13) and noncoding RNA genes by RNAmmer (14) and Rfam (15). Metabolic pathways and subcellular localization were predicted using KEGG and PSORTb, respectively (16, 17), and functional domains were evaluated using InterProScan (18).

### Accession number(s).

These whole-genome shotgun projects have been deposited in GenBank under the accession numbers listed in Table 1. We have also made the strains available to the research community by depositing them with the Biodefense and Emerging Infections (BEI) Research Resource Repository (see BEI numbers in Table 1).

**TABLE 1:** Identifiers and nucleotide sequences for sequenced strains of vaginal *Actinobacteria*

Genus/species	Strain	BEI catalog no.	Nucleotide accession no.
*Actinomyces neuii*	MJR8396A	HMS-1266	LRPJ00000000
*Alloscardovia omnicolens*	CMW7705A	HMS-1282	LRPK00000000
*Atopobium vaginae*	CMW7778A	HMS-1300	LSOA00000000
*Bifidobacterium bifidum*	MJR8628B	HMS-1264	LRPO00000000
*Bifidobacterium breve*	GED8481	HMS-1261	LRPP00000000
*Bifidobacterium longum*	CMW7750	HMS-1299	LRPQ00000000
*Corynebacterium* sp.	CMW7794	HMS-1295	LSRB00000000
*Gardnerella vaginalis*	GED7275B	HMS-1272	LRPZ00000000
*Gardnerella vaginalis*	GED7760B	HMS-1284	LRQA00000000
*Gardnerella vaginalis*	CMW7778B	HMS-1298	LSRC00000000
*Propionibacterium avidum*	MJR7694	HMS-1291	LRVD00000000
